# Overview of trials from AHA 2021

**DOI:** 10.21542/gcsp.2021.29

**Published:** 2021-12-31

**Authors:** Michael Gergis, Sherif Nagy, Robert O. Bonow

**Affiliations:** 1 Aswan Heart Centre, Cardiology Department, Aswan, Egypt; 2 Cairo University, Kasr Al Ainy Hospitals, Cardiology Department, Cairo, Egypt; 3 Division of Cardiology, Department of Medicine, Bluhm Cardiovascular Institute, Northwestern University, Chicago, Illinois, USA

## Introduction

Even though the American Heart Association Scientific Sessions 2021 was a fully virtual meeting, it caught the attention of cardiologists with its prestigious program and practice-changing sessions and late breaking clinical trials. We report on some of the top trials presented during this meeting.

### Aortic Valve Replacement versus conservative treatment in asymptomatic severe aortic stenosis (The AVATAR trial)

The outcomes of surgical aortic valve replacement (SAVR) have improved over the past decades. However, taking the decision to operate on asymptomatic severe aortic stenosis (AS) with preserved left ventricular function continues to be a matter of debate^1^**.**

The AVATAR trial was designed to challenge the current dogma that relies on symptoms and left ventricular systolic dysfunction as thresholds for intervention in the management of AS^2,3^. It was a physician-initiated, prospective, multicenter and multinational, randomized, controlled, event-driven trial that evaluated the safety and efficacy of early surgery in the treatment of asymptomatic patients with severe AS and normal LV ejection fraction (LVEF). It was noteworthy that a negative exercise test was a prerequisite for enrolment in order to recruit truly asymptomatic patients^4^.

One hundred and seventy-five (175) patients were enrolled in this study, who were randomized to either SAVR or conservative surveillance. The primary endpoint was a composite of all-cause mortality or major adverse cardiovascular events (MACE) comprised of acute myocardial infarction (AMI), stroke and unplanned heart failure (HF) hospitalization. The incidence of the primary endpoint was significantly lower in the patients randomized to SAVR compared to the conservative group (15.2% vs 34.7%) respectively, (HR= 0.46, 95% CI [0.23–0.90], *p* = 0.02).

The AVATAR results are in line with the recently published RECOVERY trial that investigated the hypothesis of early surgery for asymptomatic patients with very severe AS. ^5^ Although RECOVERY was a single center experience, it was distinguished by recruiting patients with more critical AS. However, AVATAR was designed as multicenter, multinational trial in order to recruit a broader population, with the implementation of negative stress test to ensure unmasking of symptoms, if any.

In conclusion, reading these results in the light of previously published observational data adds support for early intervention in severe AS patients, regardless of the onset of symptoms. Trials of transcatheter intervention for severe asymptomatic AS are currently in progress.

### Posterior left pericardiotomy for the prevention of atrial fibrillation after cardiac surgery (PALCAS)

Postoperative atrial fibrillation (AF) is frequently encountered following cardiac surgery^6^. It has been shown to be linked with early and late stroke, as well as worse long-term outcomes^7^. Many preventive strategies have been implemented to prevent postoperative AF. These strategies target mainly neurohormonal mechanisms using drugs acting on the sympathetic pathway, atrial conduction, and refractory periods such as beta-blockers, digoxin, and amiodarone. Furthermore, among many hypotheses of triggers of postoperative AF, pericardial effusion has been incriminated in AF pathogenesis even in the absence of frank tamponade. This can be through induction of atrial arrythmias, either via mechanical compression or by promoting inflammation and oxidative stress^8^. Given its high incidence following cardiac surgery^9^, pericardial effusion has recently been introduced as a target to prevent postoperative AF.

The PALCAS trial investigated the hypothesis of decreasing the incidence of postoperative AF by continuous drainage of pericardial fluid to the pleural cavity by performing a posterior pericardial incision^10^. This was a randomized, single-center study that included patients undergoing elective primary cardiac surgery on coronary arteries, aortic valve, and ascending aorta or combinations of these. In contrast, patients undergoing mitral, and tricuspid valve surgery and those with a history of AF were excluded^11^.

The trial enrolled 420 adult patients who were randomized to undergo either posterior left pericardiotomy (212) or no intervention (208) other than the planned surgical procedure. Furthermore, patients were stratified by the CHA_2_DS_2_-VASc score (score of ≤2 vs ≥3), to guarantee similar risk of postoperative AF^12^. Both patients and assessors were blinded to treatment group assignment.

**Figure 1.**
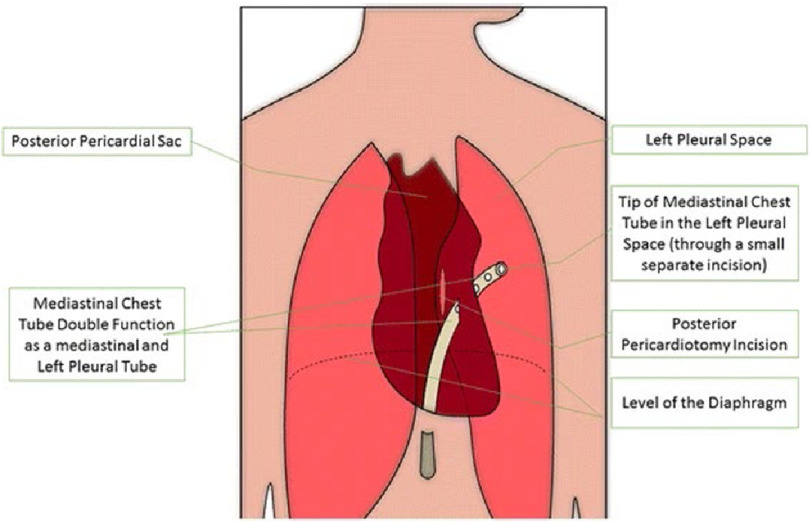
Double function of the mediastinal chest tube in posterior pericardiotomy patients. Reproduced under CC BY licence from https://doi.org/10.1186/s13063-017-2334-4.

However, as posterior pericardiotomy requires opening of the left-sided pleura and positioning of the tip of the mediastinal tube in the left-sided thorax, patients in the intervention group were distinguishable from those in the control group who may or may not have a left-sided pleural tube in situ. For those patients in the intervention group, the standard mediastinal chest tube served doubly functional as a mediastinal tube and a left-sided pleural tube (Figure 1). This resulted in a single-blinded study, with the patients not knowing to which arm of the study they were assigned.

The primary outcome was the occurrence of in-hospital postoperative AF lasting ≥30 s. Safety outcomes were operative mortality, postoperative major adverse events (defined as all-cause mortality, stroke, and myocardial infarction), and postoperative clinical or imaging evidence of left pleural or pericardial effusion.

The two groups were similar with respect to baseline clinical and surgical characteristics. The incidence of postoperative AF was lower in the intervention group vs conservative group (17% vs 32%); *p* = 0.0007. Odds ratio adjusted for the stratification variable 0.44 [95% CI [0.27–0.70]; *p* = 0.0005]. The incidence of postoperative pericardial effusion was higher in the conservative than in the intervention group (21% vs 12%). Interestingly, there was no difference in the incidence of left pleural effusion between the two groups. Postoperative beta blockers were given in 95% and 90% of intervention and control groups, respectively. When limiting the analysis to those receiving beta blockers, AF occurred in 11% in intervention group vs 26% in controls. No posterior left pericardiotomy related complications were seen as per treated analysis.

In conclusion, this adequately powered randomized trial shows significant reduction in postoperative AF by performing posterior left pericardiotomy. Moreover, this simple procedure added minimal time to the operation time with no reported complications.

The study has some limitations. Firstly, it is single-center experience. Secondly, it recruited patients with relatively low risk of postoperative AF by excluding those undergoing mitral or tricuspid valve surgery or with a history of previous atrial arrhythmias. Thirdly, is the relatively small sample size. Further confirmation of these results in larger, multi-center studies is warranted.

### The Empagliflozin Outcome Trial in Patients with Chronic Heart Failure with Preserved Ejection Fraction (EMPEROR-Preserved trial)

Sodium–glucose co-transporter 2 (SGLT2) inhibitors, that initially emerged as a new diabetic treatment, succeeded to establish their role as one of the four pillars for the management of heart failure with reduced ejection fraction (HFrEF)^13^. Heart failure with preserved ejection fraction (HFpEF) continues to be a complex condition lacking effective evidence-based treatment for many years, thus creating the opportunity to investigate effectiveness of SGLT2 inhibitors in HFpEF.

Recently, the safety and efficacy of SGLT2 inhibitors in HFpEF was tested in the EMPEROR-Preserved trial. It was a randomized, multicenter, double-blinded, placebo-controlled trial that evaluated the effects of the SGLT2 inhibitor empagliflozin on major heart failure outcomes in patients with HFpEF^14^.

The study enrolled 5,988 patients with symptomatic heart failure and preserved ejection fraction (EF 40%). The patients were randomized in a 1:1 fashion into two groups, to receive either empagliflozin (10 mg per day, *n* = 2,997) or placebo (*n* = 2,991) in addition to usual therapy. Follow up was done for median duration of 26.2 months for symptoms, health status (assessed with the Kansas City Cardiomyopathy Questionnaire [KCCQ]), and adverse events.

**Figure 2.**
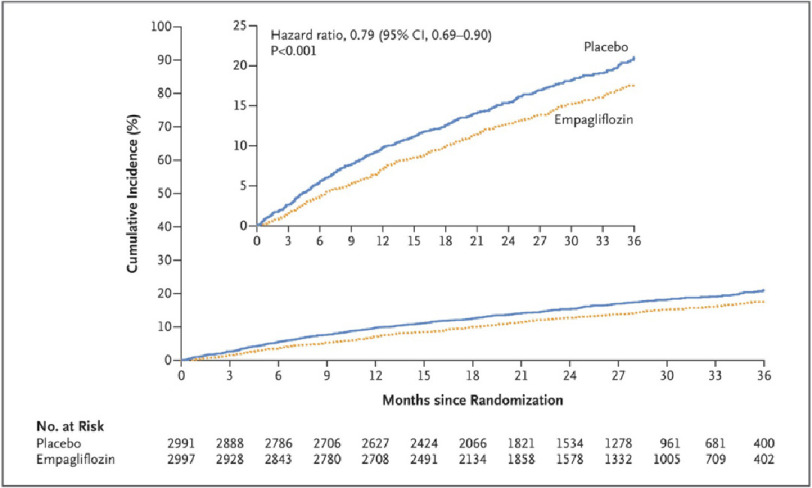
Estimated cumulative incidence of the primary outcome (composite of cardiovascular death or hospitalization for HF) in the empagliflozin group compared to the placebo group. N Engl J Med. 2021 Oct 14;385(16):1451–1461 DOI: 10.1056/nejmoa2107038.

The primary composite outcome [cardiovascular (CV) death or hospitalization for heart failure (HHF)] was significantly lower in the empagliflozin group compared to the placebo group (13.8% vs 17.1%), (HR, 0.79; 95% CI [0.69–0.90]; *P* < 0.001), with the number needed to treat was 31 (95% CI [20–69]) (Figure 2).

The reduction in primary outcome was driven mainly by reduction in HHF (8.6% vs 11.8%), HR, 0.71; 95% CI, 0.60 to 0.83). However, there was no significant difference with respect to CV death (7.3% vs 8.2%). Regarding the secondary outcome, the total number of HHF was lower with empagliflozin than with placebo (hazard ratio, 0.73; 95% CI, 0.61 to 0.88; *P* < 0.001), and the rate of decline in the estimated glomerular filtration rate was slower in the empagliflozin group than in the placebo group (−1.25 vs. −2.62 ml per minute per 1.73 m^2^ per year; *P* < 0.001).

In conclusion, in patients with HFpEF, empagliflozin led to a 21% risk reduction of the composite of CV death or HHF, which was mainly related to a 29% lower risk of HHF, rather than any significant effect on CV death. This effect was consistently observed across all pre-specified subgroups, for all patients with or without diabetes. Empagliflozin also led to a longer time to first HHF. The door is open now for more trials of other members of this prestigious anti-diabetic group investigating their role in HFpEF.

### Sodium–glucose co-transporter 2 inhibition in patients hospitalized for acute decompensated heart failure (EMPULSE trial)

As noted above, the SGLT2 inhibitors represent a breakthrough in the management of heart failure (HF) by reducing the CV death and HHF^15,16^. This benefit is evident across the whole spectrum of left ventricular ejection fraction (LVEF). With previously shown efficacy for stable chronic HFrEF, both in patients with and without type 2 diabetes in the two large, randomized trials (DAPA-HF^17^ and EMPEROR-Reduced^18^).

Moreover, its efficacy has also now been shown in patients with HFpEF in the EMPEROR-Preserved trial^19^. The SOLOIST-WHF trial expanded the evidence of reducing death and HHF in semi acute HF patients^20^, but this was limited to diabetic patients. Whether in-hospital initiation leads to clinical benefit and is safe in patients with and without diabetes and irrespective of LVEF, remained a question to be answered.

**Figure 3.**
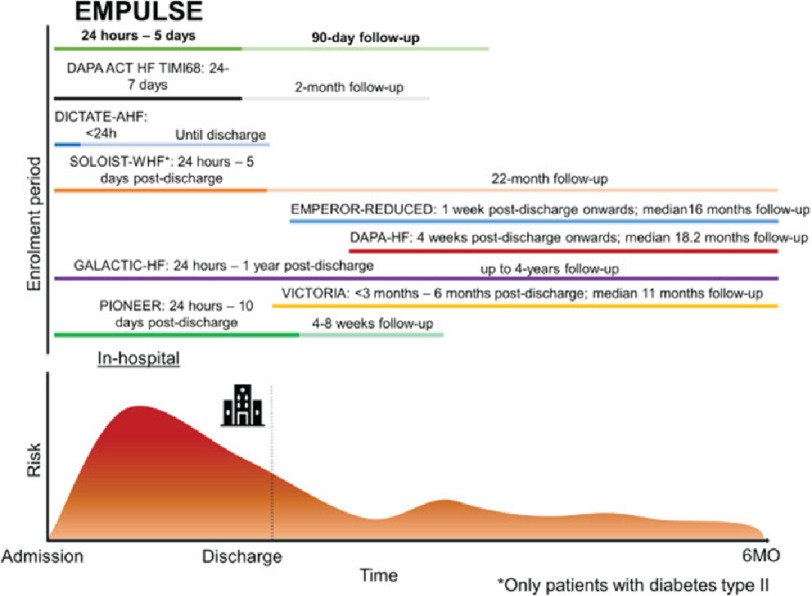
Timeline of EMPULSE for enrolment vs. timelines of enrolment for other recent trials targeting patients with heart failure. The timeline for follow-up is shown in lighter color. When follow-up and inclusion overlap, a darker color is used. Eur. J. Heart Fail. 23, 826–834 (2021).

EMPULSE was thus a highly-awaited trial investigating the efficacy and safety of empagliflozin in patients hospitalized with acute heart failure, whether they had type 2 diabetes mellitus (T2DM) or not, and HF irrespective of LVEF, thus providing the missing link in the continuum of care between HFrEF and HFpEF trials. See Figure 3.

EMPULSE was a multinational, multicenter, randomized, double-blind superiority trial to evaluate the effects of once daily oral empagliflozin 10 mg compared to placebo on clinical benefit, safety, and tolerability in patients hospitalized for acute HF after initial stabilization^21^. The patients were enrolled after the primary diagnosis of acute HF and high levels of natriuretic peptides irrespective of LVEF and with or without T2DM.

The randomization was done after initial stabilization between 24 h and 5 days after admission. The study excluded patients in cardiogenic shock, acute myocardial infarction (MI), low estimated glomerular filtration rate (eGFR) below 20 ml/min/1.73 m^2^, major cardiac surgery, type I DM, and current or prior treatment with SGLT1 or SGLT2 inhibitors in the 90 days prior to enrolment.

Patients were also stratified as de-novo HF or worsening chronic HF. The primary outcome was clinical benefit at 90 days, defined as time to all-cause death, the number of HF events (HFE), time to first HFE and a ≥5 point increase from baseline in KCCQ total symptom score (KCCQ-TSS) after 90 days of treatment, assessed using a “win-ratio” approach.

The study has unique aspects in the design of its inclusion window and duration of follow up, also using the win-ratio approach in the statistical analysis. The win ratio is an analysis of composite endpoints in clinical trials based on clinical priorities^22^, in which each patient in the trial is compared to every other patient within each stratum (new-onset HF vs. decompensated chronic HF) in a pairwise hierarchical fashion. The win ratio was calculated as the total number of wins in the empagliflozin group across all strata divided by the total number of losses.

The trial enrolled 530 patients randomized in 1:1 ratio, of whom 33% had de-novo acute HF, 45.3% had DM, the mean baseline EF was 35.1% and 31.9% patients had LVEF >40%. Patients treated with empagliflozin were 36% more likely to experience clinical benefit (win-ratio: 1.36, *P* = 0.0054) and this benefit was consistent across all the spectrum of primary outcome.

Regarding the individual components of the primary endpoint, there was 35% reduction in death and HFE in the empagliflozin group; *P* = 0.04. Fewer patients had serious adverse events in the treatment group (32.3% vs 43.6%), and acute renal failure occurred more in the placebo group. No episodes of diabetic ketoacidosis occurred.

In conclusion, empagliflozin initiation in hospitalized patients with acute HF after initial stabilization was well tolerated and showed significant clinical benefit within 90 days, significant improvement in quality of life and reduction in natriuretic peptides.

### Canagliflozin: Impact on Health Status, Quality of Life and Functional Status in Heart Failure (CHIEF-HF trial)

Large clinical trials are costly, with data collection accounting for almost half of the expense. The dismal implications of the COVID-19 pandemic added more restrictions on physical patient visits in the context of clinical trials. How can clinical trial participants be enrolled amid these unprecedented circumstances and restrictions? The CHIEF-HF trial aimed to answer this question.

The CHIEF-HF trial was the first trial to be conducted 100% virtually, where participants were engaged without in-person contact. The idea of this study was to use patient-reported response as an outcome, thanks to widely-available mobile technology.

The CHIEF-HF trial was designed with the primary outcome of the KCCQ-TSS to capture the benefits of canagliflozin over placebo on patients’ shortness of breath, fatigue, orthopnea, and edema ^23^.

Recently, the KCCQ was approved by the Food and Drug Administration’s Center for Drug Evaluation and Research as a clinical outcome assessment tool for drug approval^24^. It was introduced in different SGLT2 inhibitors trials (such as DAPA-HF and EMPEROR-reduced) where improvement in HF symptoms was measured by the KCCQ^17,18^.

The patients eligible for CHIEF-HF were adults aged ≥ 18 years with the diagnosis of HF irrespective of LVEF or presence or absence of T2DM. Patients used their own smart phones to support the mobile data collection of KCCQ during the 3-month period of active medication and were also encouraged to wear a Fitbit Versa 2 for 9 months from randomization to monitor activities. Key exclusion criteria were; concurrent use of SGLT2 inhibitors, history of diabetic ketoacidosis, T1DM, and estimated glomerular filtration rate <30 ml/min/1.73 m^2^.

This unique design of eliminating the physical onsite visits enabled the study to launch in the midst of the COVID-19 pandemic. It was a randomized, double-blind, placebo-controlled, decentralized, interventional, superiority study. After virtual informed consent and KCCQ ≤ 80, the patients were randomized to either canagliflozin 100 mg daily (*n* = 222) or placebo (*n* = 226) for 3 months followed by unblinding then data collection for another 6 months.

Nearly 60% had HFpEF defined as EF >40% and 28% had DM. After 3 months, the KCCQ was better by mean 4.3 points in the active treatment group (*p* = 0.016) and this was similar irrespective the type of HF or DM status. Regarding the secondary outcome, all-cause mortality was 0.9% in active group vs 1.7% (*p* > 0.05).

In conclusion, canagliflozin improved HF symptoms in patients with or without DM and across the spectrum of LVEF. This trial design opens the door for conducting more virtual trials where the outcomes could be monitored and/or assessed by smart technology.

### Antithrombotic Treatment with Factor XIa Inhibition to Optimize Management of Acute Thromboembolic Events in Total Knee Replacement [AXIOMATIC-TKR])

Achieving balance between effective anticoagulation and low bleeding risk continues to be an area of struggle for physicians. In this context, direct oral anticoagulants (DOACs) are expanding to replace vitamin K antagonists for many indications. However, bleeding as a side effect continues to cause some physicians to prescribe lower doses of DOACs. Recent animal-based studies introduced factor XI as a new target for safer anticoagulation with data showing factor XI deficiency protected against thrombosis with negligible bleeding risk ^25^.

Milvexian was recently introduced as a bioavailable oral molecule with selective factor XIa inhibition at high affinity with a half-life of approximately 12 h ^26^. It was recently shown in the Axiomatic-TKR trial to achieve significant reduction in venous thromboembolism (VTE) compared to enoxaparin, with similar bleeding events in both arms^27^.

The Axiomatic TKR trial was presented at AHA 2021 as a proof-of-principle phase-2 trial, comparing the efficacy and safety of milvexian and enoxaparin in patients undergoing elective knee arthroplasty. It included medically stable patients ≥50 years of age who were appropriate candidates for anticoagulant prophylaxis. The main exclusion criteria were contraindications to enoxaparin, history of severe hepatic impairment or previous venous thromboembolism (VTE), the use of long-term antithrombotic therapy other than aspirin (≤100 mg per day), or the inability to undergo venography.

It was designed as randomized, parallel-group trial with open label for treatment assignment to milvexian or enoxaparin. However, patients and observers were blinded to milvexian dose. Eligible patients were randomly assigned to 1 of 7 parallel treatment groups included four regimens of twice daily milvexian (25 mg, 50 mg, 100 mg, or 200 mg), two regimens of once daily milvexian (25 mg or 200 mg), and enoxaparin (40 mg once daily), respectively. Trial medications were started 12–24 h postoperatively and continued for 10–14 days.

The dosing regimen of 25 mg was stopped early, based on *ad hoc* pre-specified interim analysis that showed an insufficient efficacy. Subsequently, another regimen (50 mg once daily) was added. The primary outcome was a composite of asymptomatic deep-vein thrombosis (DVT) confirmed by unilateral mandatory venography 10–14 days after surgery, confirmed symptomatic VTE, or death from any cause. The major secondary efficacy outcomes were proximal or distal DVT (symptomatic or asymptomatic), nonfatal pulmonary embolism, and death.

After enrollment of 1,242 subjects randomized from 118 centers in 18 countries. The overall rates of VTE in the milvexian arm at any given dose was significantly lower than the enoxaparin arm, including the prespecified safety benchmark (12.2%) vs (30%) with the latter representing the VTE rate among patients undergoing surgery without thromboprophylaxis. Notably, the dose response relationship was significant at both once and twice daily doses of milvexian when compared to enoxaparin (*p* = 0.0003 and *p* = 0.0004, respectively). Moreover, the secondary proof of efficacy for milvexian was met by achieving lower rates of VTE when compared to enoxaparin with total daily doses of 100 mg milvexian or greater. Regarding safety outcomes, there was no significant difference between both groups. bleeding was 4% with both milvexian and enoxaparin.

In conclusion, milvexian factor XIa inhibitor was effective for preventing venous thromboembolism and was associated with a low risk of clinically relevant bleeding. While, Axiomatic-TKR is a phase 2 trial and more definitive trials are needed, it does represent a step forward to finding a safer oral anticoagulant in the near future.
